# Glycosylated hemoglobin A1c predicts coronary artery disease in non‐diabetic patients

**DOI:** 10.1002/jcla.23612

**Published:** 2020-10-09

**Authors:** Yildiz Kayali, Aclan Ozder

**Affiliations:** ^1^ Medical Faculty Department of Family Medicine Bezmialem Vakif University Istanbul Turkey

**Keywords:** coronary artery disease, hemoglobin A1c, non‐diabetic, prediabetic, primary care

## Abstract

**Background:**

In primary care, there is a need for simple and cost‐effective tool that will allow the determination of the risk of coronary artery disease (CAD). We aimed to research the value of glycosylated hemoglobin (HbA1c) in the prediction of coronary artery disease.

**Methods:**

Patients admitted to the outpatient clinic of the Cardiology for angiography were retrospectively screened. Patients with diabetes or with HbA1c of 6.5 or above were excluded. Comparative HbA1c data were obtained according to the stenosis groups. Logistic regression analysis was used to investigate the risk factors affecting stenosis positivity.

**Results:**

Of the study group, 120 patients were without any stenosis in any coronary artery, 56 patients were with >50% stenosis in one coronary artery, and 71 patients were with >50% stenosis in more than one coronary artery. There was a statistically significant difference between HbA1c measurements according to the degree of stenosis (*P* = .001 and *P* < .01, respectively). The odd ratio for HbA1c was 6.260 (95% CI: 3,160‐12,401). According to the stenosis positivity, the cutoff point for HbA1c was found to be 5.6 and above. In the regression analysis, HbA1c was an independent risk factor for CAD. One unit increase in HbA1c level increases the risk of stenosis up to 12.4‐fold (95% CI: 5,990‐25,767).

**Conclusion:**

The study showed HbA1c can be used as an independent marker in determining the probability and severity of coronary artery disease in non‐diabetic individuals and as a useful marker in primary care predicting CAD.

## 1 INTRODUCTION

Coronary artery disease (CAD) is one of the leading causes of morbidity and mortality worldwide.^1^ CAD remains as a leading reason for heart failure causing disability and not only outcomes in the need for life‐long therapies but also causes sudden death with acute myocardial infarction.^2^ CAD turns to be the first leading cause of death in Turkey.^3^

In terms of CAD, the goal of preventive medicine is to provide healthier and longer lifespan with lower costs. For this reason, there is a need for biochemical markers that will enable the determination of the risk of CAD in preventive medicine.

Diabetes mellitus is a common metabolic disorder characterized by absolute or relative deficiencies in insulin secretion and/or insulin action associated with chronic hyperglycemia and with an increased risk of microvascular and macrovascular disease.^4^ Having seen the results of the Diabetes Control and Complications Trial (DCCT), the medical community devoted their attention to the significance of keeping blood glucose levels within normal range as much as possible in order to prevent or delay the severe and serious complications of type 2 diabetes mellitus (T2DM).^5^

It is essential to achieve glycaemic control in diabetes for better prognosis of the disease. The previous studies in the literature revealed that maintaining the metabolic control has a profound impact on retarding long‐term complications of diabetes and even preventing them.^6^, ^7^

However, the accurate mechanisms remain unclear, it was shown that CAD is the result of an interactive relation between environmental and genetic factors in epidemiological studies. Hypertension, hypercholesterolemia, diabetes mellitus, smoking, age, and gender are responsible risk factors for the occurrence of CAD.^8^

In recent studies, it was reported that atherosclerotic vessel disease is a slow and insidious process with inflammation and lipid accumulation in principal and occurs with the adverse effects of various risk factors. A plaque is formed inside the vessel during the ongoing process. The present amount of lipids and necrotic and apoptotic cells, the fibrous structure, and the level of inflammation and neovascularization determine the vulnerability of the plaque to be ruptured. Acute coronary syndrome develops with the rupture of the plaque. The goal of the primary care is to prevent atherosclerotic plaque formation and thus acute coronary syndrome.^9^

Glycosylated hemoglobin A1c (HbA1c) is a marker of 3‐month blood sugar levels. Long‐term high blood sugar levels have been shown to pose a risk of cardiovascular disease.^10^ The studies have shown that high HbA1c value in diabetes patients leads to an increase in overall mortality and cardiovascular disease mortality.^11^, ^12^ In addition, it has been shown that HbA1c may be a marker for determining the severity of cardiovascular disease in patients with diabetes.^13^, ^14^

Several studies have examined the relationship between HbA1c and CAD. However, while some of these studies were performed on diabetic patients, significant relationships were not found when independent risk factors were removed.^15^, ^16^

The aim of this study was to explore a laboratory criterion that can be used to diagnose CAD early in subjects with pre‐diabetes which can be applied widely even in outpatient conditions. In this context, we aimed to question whether HbA1c molecule may be an independent predictor of CAD.

## 2 MATERIALS AND METHODS

### 2.1 Design, setting, and sample

The study was done among 247 patients without T2DM who were admitted to the outpatient clinic of the Cardiology department from January 2016 to June 2018 in a university hospital. The non‐diabetic patients with HbA1c value below 6.5% who underwent elective coronary angiography (CAG) with various indications were included in the study. The age range of participants was 30‐90 years.

The study group was divided into groups according to stenosis degrees yielded from elective angiograms that showed (a) stenosis of 50% or more in only one of the main branches of the left main coronary artery or left anterior descending artery or left circumflex artery or right main coronary artery and coronary vascular system, (b) more than 50% stenosis in more than one coronary arteries, and (c) the patients with normal coronary vessels.

### 2.2 Measurements

In addition to obtaining HbA1c results of all patients included in the study, other biochemical tests performed before angiography including high‐density lipoprotein (HDL), low‐density lipoprotein (LDL), triglyceride (TG), liver function tests (AST, ALT), and electrolytes (Na, K, Ca). Demographic characteristics and known chronic diseases were investigated by face‐to‐face interviews. The study was approved by Institutional Review Board at the Bezmialem Vakif University, and the patients provided written informed consent to participate in the study.

### 2.3 Definitions

The patients were considered having diabetes mellitus (DM) when HbA1c was >6.5%.^16^ Patients with DM, with anti‐diabetic medication for any reason, who had an active infection, hemoglobinopathy, myocardial infarction with active ST elevation, and giving a history of coronary bypass surgery (CABG) were excluded.

### 2.4 Data analysis

NCSS (Number Cruncher Statistical System) 2007 (Kaysville) was used for statistical analysis. While evaluating the study data, the Student t test was used for comparison of two groups of variables showing normal distribution in the comparison of descriptive statistical methods and quantitative data. One‐way ANOVA test and Bonferroni test were used for comparison of groups with the normal distribution of three and more; Kruskal‐Wallis test and Bonferroni‐Dunn test were used for comparisons between groups of three and above, which were not normally distributed. Logistic regression analysis (Backward Stepwise) was used to investigate the risk factors affecting stenosis positivity. Pearson chi‐square test and Fisher‐Freeman‐Halton test were used to compare the qualitative data. Significance was set at *P* < .05.

## 3 RESULTS

### 3.1 Demographic features of the study group

The study was conducted with a total of 247 cases whose 41.7% (n = 103) were women. The ages of the patients ranged from 33 to 87 years with a mean age of 59.96 ± 10.89 years.

### 3.2 CAG characteristics of the study group

CAG results showed that stenosis was lower than 50% in 48.6% (n = 120), higher than 50% in single coronary vascular and 28.7% (22.7%). n = 71) greater than 50% in multiple coronary vascular. In 48.6% (n = 120) of the cases, stenosis was (−), and in 51.4% (n = 127), stenosis was (+). Stenosis degrees according to descriptive characteristics are shown in Table 1.

**TABLE 1:** Evaluation of degree of stenosis according to descriptive characteristics

		Degree of stenosis			*P*
		<%50 (n = 120)	Single >%50 (n = 56)	Multiple >%50 (n = 71)	
Age (y)	Min‐Max (Median)	33‐87 (56.5)	35‐87 (60)	40‐85 (63)	^a^.003**
	mean ± SD	57.73 ± 9.97	60.70 ± 10.98	63.14 ± 11.56	
Sex n (%)	Female	64 (62.1)	22 (21.4)	17 (16.5)	^b^.001**
	Male	56 (38.9)	34 (23.6)	54 (37.5)	
HT n (%)	No	53 (52.5)	20 (19.8)	28 (27.7)	^b^.545
	Yes	67 (45.8)	36 (24.7)	43 (29.5)	
CAD n (%)	No	101 (58.7)	35 (20.4)	36 (20.9)	^b^.001**
	Yes	19 (25.3)	21 (28.0)	35 (46.7)	
Hypothyroids n (%)	No	108 (47.4)	50 (21.9)	70 (30.7)	^c^.043*
	Yes	12 (63.2)	6 (31.6)	1 (5.2)	
COPD/Asthma n (%)	No	115 (49.1)	51 (21.8)	68 (29.1)	^c^.412
	Yes	5 (38.5)	5 (38.5)	3 (23.0)	
Habit n (%)	No	82 (51.3)	33 (20.6)	45 (28.1)	^b^.457
	Yes	38 (43.7)	23 (26.4)	26 (29.9)	

^a^ One‐way ANOVA test.

^b^ Pearson chi‐square test.

^c^ Fisher‐Freeman‐Halton test.

* *P* < .05.

** *P* < .01.

### 3.3 Laboratory features of the study group

In the study, HbA1c measurements ranged from 4.48 to 6.49 (5.70 ± 0.54) in whole study group and were lower than 5.69 among 44.5% (n = 110) of the cases, and however, it was between 5.7 and 6.5 in 55.5%. (n = 137) (Table 2).

**TABLE 2:** Evaluation of HbA1c measurements according to the degree of stenosis

		HbA1c (%)		*P*
		Min‐Max (Median)	Mean ± SD	
Degree of stenosis	<%50	4.48‐6.48 (5.48)	5.41 ± 0.55	^a^.001*
	Single >%50	4.90‐6.49 (5.80)	5.84 ± 0.33	
	Multiple >%50	5.16‐6.49 (6.20)	6.06 ± 0.36	
	Stenosis (−)	4.48‐6.48 (5.48)	5.41 ± 0.55	^b^.001*
	Stenosis (+)	4.90‐6.49 (6.00)	5.97 ± 0.37	

^a^ One‐way ANOVA test.

^b^ Student's *t* test.

* *P* < .01.

### 3.4 Correlations between the HbA1c and stenosis

Stenosis (+) ratio was found to be significantly higher in cases with HbA1c level of 5.7‐6.5 than those with HbA1c level of <5.69 (*P* = .001; *P* < .01). HbA1c level 5.7‐6.5 increases the risk of stenosis 6,260 times. The odd ratio for HbA1c was 6.260 (95% CI: 3,160‐12,401) (Table 3).

**TABLE 3:** Evaluation of HbA1c levels according to stenosis positivity in patients without CAD (n = 172)

		Stenosis Situation		*P* ^a^	Odds ratio	%95 CI
		Stenosis (−)	Stenosis (+)			
		n (%)	n (%)			
HbA1c (%)	<5.69	67 (79.8)	17 (20.2)	.001*	6,260	3,160‐12,401
	5.7‐6.5	34 (38.6)	54 (61.4)			

^a^ Pearson chi‐square test.

* *P* < .01.

### 3.5 Characteristics of ROC curve regarding level of HbA1c related to positivity of stenosis

**FIGURE 1:**
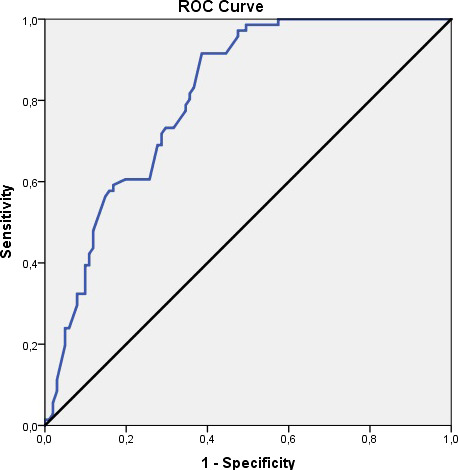
ROC curve regarding level of HbA1c related to positivity of stenosis

According to the stenosis positivity, the cutoff point for HbA1c was found to be 5.6 and above. HbA1c for cutoff value of 5,6; sensitivity 91.55%; specificity 61.39%; positive predictive value was 62.50%, and negative predictive value was 91.18%. In the obtained ROC curve, 80.9% standard error was 3.2% (Figure 1).

A statistically significant correlation was found between the presence of stenosis and the cutoff value of HbA1c level 5,6 (*P* = .001; *P* < .01). The risk of stenosis is 17.2 times higher in cases with HbA1c level of 5.6 and above. The odds ratio for HbA1c is 17.2 (95% CI: 6,814‐43,530).

The variables included in the study were included in logistic regression analysis, 4 step end of study: age, sex, habit (smoking and alcohol), and HbA1c measurements, which are among the risk factors that affect stenosis positivity, are a significant model. The explanatory coefficient of the model is 74.9%. One unit increase in HbA1c measurements increases the risk of stenosis positive to 13,177 times (95% CI: 6,283‐27,634). In conclusion, age, sex, habit, and HbA1c are independent risk factors.

## 4 DISCUSSION

In this retrospective, single‐center study of non‐diabetic patients, between 30 and 90 years of age, we examined prevalence and severity of CAD and their association with HbA1c and CAD risk factors. Further, we examined the relationship of demographic and CAD risk factors and HbA1c to coronary plaque composition in a fashion that has not been previously studied in primary care in Turkey to the best of our knowledge.

HbA1c is one of the recommended parameters in follow‐up of diabetic patients, showing a mean plasma glucose level of recent 3 months. HbA1c level is an important risk indicator for microvascular complications of diabetes, and it is well known that higher HbA1c is a potential indicator for death in acute coronary syndrome (ACS) patients and a predictor for short‐term mortality in ACS patients without DM^16^; however, it is not yet clear whether to use it as a risk indicator for macrovascular complications.^17^

The relationship between HbA1c and CAD has been studied to be clarified in many studies over the years, and a certain number of data accumulation has been achieved. The revealed data showed there is a significant relationship between HbA1c and CAD in recent studies in the literature.^18^, ^19^, ^20^ In our study, as in line with previous studies, there was a strong relationship between HbA1c level and CAD in non‐diabetic patients indicating HbA1c could be used as a predictor to determine coronary atherosclerosis (*P* < .01).

In a study in the literature with 292 patients, it was aimed to correlate the severity of coronary artery disease with HbA1c value, and however, it was found no significant relationship between GENSINI score and HbA1c; thus, it is stated that HbA1c cannot be used as an independent marker for CAD severity.^21^ However, in another study, it was showed that HbA1c level correlated positively with GENSINI score in diabetic and non‐diabetic population.^22^ In a prospective study in India, a SYNTAX score was used to determine the severity of coronary artery disease, and it was found a high correlation between HbA1c levels and severity of coronary artery disease and number of diseased vessels and increased SYNTAX score in the non‐diabetic population.^23^ In our study, a statistically significant difference was found between severity of CAD and HbA1c measurements (*P* < .01).

Although there were found contrary results in many studies, in a published meta‐analysis of 27 prospective studies in Western societies, of them nine were contributed to the relationship between HbA1c and CAD. As a result, a 1.2‐fold increase in CAD was reported for each 1% increase in HbA1c in non‐diabetics. In the same meta‐analysis, it was reported that fasting and post‐prandial glucose levels were significantly associated with CAD risk in non‐diabetic patients; however, the relationship between HbA1c and CAD risk was slightly stronger.^24^ In a prospective study conducted in 93 patients, the relationship between the severity of coronary artery disease and HbA1c levels was evaluated by logistic regression analysis and higher levels of HbA1c were determined as independent predictors of severe atherosclerosis.^25^

A logistic regression model including age, gender, hypertension, hypothyroidism, hyperlipidemia, smoking habit, and HbA1c showed that only HbA1c (*P* < .01) was associated with CAD. Age was associated with a more than 1.04‐fold (%95 CI: 1,014‐1,080), gender with a more than 2.94‐fold (95% CI: 1,527‐5,676), as was smoking with a more than 2.05‐fold (95% CI: 1,009‐4,155) increased risk of CAD in an adjusted logistic regression model. Our findings are consistent with other studies in the literature.^26^, ^27^ We detected one unit increase in HbA1c level was associated with an increase in the risk of CAD 13.2‐fold (95% CI: 6,283‐27,634). The difference in our findings in HbA1c from previous studies^24^, ^25^ may be due to HbA1c level is associated with several other traditional cardiovascular risk factors such as genetic expression, physical inactivity, and dietary imbalance.

Previous epidemiologic studies have demonstrated a significant association between coronary atherosclerosis and gender.^28^, ^29^ The current study was able to further expand these prior findings by assessing the relationship of sex and CAD to the prevalence and severity of coronary atherosclerosis. In our sample, 37.5% of the male study subjects (n = 54) and 16.5% of the female study subjects (n = 17) were with stenosis in multiple vessels (*P* < .01). This prevalence was similar to the results of some studies with the rates ranging 21%‐60% among males and ranging 12%‐40% among females in the general population.^30^, ^31^ In this regard, we identified male gender as a strong predictor of coronary atherosclerosis.

In a study conducted, the optimal cutoff value for the prediction of HbA1c in severe CAD in ROC curve with 74.4% sensitivity and 75.1% specificity was 6.52%.^10^ In another study with 299 patients, GENSINI score was used to determine the severity of CAD and a significant relationship was found with HbA1c. The ideal cutoff value of HbA1c for the emerging coronary artery disease was found to be 5.6%.^32^ In a previous study with 411 patients in recent years, based on the ROC curve, the cutoff value of HbA1c between patients with and without coronary atherosclerosis was found to be 5.45%.^33^ In our study, ROC analysis was used and HbA1c cutoff value with 91.55% sensitivity and 61.39% specificity was found as 5.6% in determining atherosclerotic patients. We also showed that the increase in HbA1c value is related to the number of diseased vessels (*P* < .01).

The lower limit of HbA1c to diagnose pre‐diabetes is 5.7 in the modern medicine.^34^, ^35^ The results of our study show that the risk for coronary artery disease increases significantly at HbA1c levels of 5.6 and above. We concluded that the risk of stenosis is 17.2‐fold higher in cases with HbA1c level of 5.6 and above. Therefore, we thought that paying attention to pre‐diabetics will provide an advantage in the early diagnosis of risky atherosclerotic conditions in preventive medicine.

The inconsistencies between HbA1c and coronary artery disease correlations in our examples compared to the literature may be due to the facts that the studies were done in different ethnic groups, different study methods, different scoring systems in CAD grading, and different HbA1c measurement methods. There is a need for a larger number of studies that take these conditions into account in order to conclusively prove that HbA1c can be used as a predictor of coronary artery disease in patients without diabetes.

Some limitations of our study need to be addressed and the results should be used more carefully for other ethnic groups as it is performed only in a population limited to Turkish patients. In addition, our study is a relatively small scale study from a single center. HbA1c promises to have value in identifying angiographic CAD in non‐diabetic individuals but would need further prospective research with larger patient population.

In our study, we demonstrated that HbA1c can be used as an independent marker in determining the severity of coronary artery disease in non‐diabetic individuals. We think that this result can give an idea in preventive medicine in terms of which patients are at risk and/or should be taken into preventive healthcare procedures. As a result of this study, we planned to offer to family physicians to check HbA1c levels among their patients in primary care and to consider a cardiological follow‐up to individuals with increased risk. In this way, we think that we can slow down the course of CAD and prevent worse outcomes.
